# Trends in Hepatocellular Carcinoma Incidence and Risk Among Persons With HIV in the US and Canada, 1996-2015

**DOI:** 10.1001/jamanetworkopen.2020.37512

**Published:** 2021-02-17

**Authors:** Jing Sun, Keri N. Althoff, Yuezhou Jing, Michael A. Horberg, Kate Buchacz, M. John Gill, Amy C. Justice, Charles S. Rabkin, James J. Goedert, Keith Sigel, Edward Cachay, Lesley Park, Joseph K. Lim, H. Nina Kim, Vincent Lo Re, Richard Moore, Timothy Sterling, Marion G. Peters, Chad J. Achenbach, Michael Silverberg, Jennifer E. Thorne, Angel M. Mayor, Heidi M. Crane, Mari M. Kitahata, Marina Klein, Gregory D. Kirk

**Affiliations:** 1 Department of Epidemiology, Johns Hopkins University Bloomberg School of Public Health, Baltimore, Maryland; 2 Kaiser Permanente Mid-Atlantic States, Mid-Atlantic Permanente Research Institute, Rockville, Maryland; 3 Division of HIV/AIDS Prevention, Centers for Disease Control and Prevention, Atlanta, Georgia; 4 Department of Medicine, University of Calgary, Calgary, Alberta, Canada; 5 Department of Medicine, Yale University, West Haven, Connecticut; 6 National Cancer Institute, Bethesda, Maryland; 7 Division of General Internal Medicine, Icahn School of Medicine at Mount Sinai, New York, New York; 8 Division of Infectious Diseases, Icahn School of Medicine at Mount Sinai, New York, New York; 9 Department of Medicine, University of California, San Diego, San Diego; 10 Center for Population Health Sciences, Stanford University School of Medicine, Stanford, California; 11 Department of Medicine, Yale University School of Medicine, New Haven, Connecticut; 12 Department of Medicine, University of Washington, Seattle; 13 Center for Clinical Epidemiology and Biostatistics, Perelman School of Medicine, Department of Biostatistics, Epidemiology, and Informatics, University of Pennsylvania, Philadelphia; 14 Center for Pharmacoepidemiology Research and Training, Perelman School of Medicine, Department of Biostatistics, Epidemiology, and Informatics, University of Pennsylvania, Philadelphia; 15 Perelman School of Medicine, Department of Medicine, University of Pennsylvania, Philadelphia; 16 Department of Medicine, Johns Hopkins University, Baltimore, Maryland; 17 Department of Medicine, Vanderbilt University School of Medicine, Nashville, Tennessee; 18 Department of Medicine, University of California, San Francisco, San Francisco; 19 Division of Infectious Diseases, Northwestern University Feinberg School of Medicine, Chicago, Illinois; 20 Kaiser Permanente Northern California, Oakland; 21 Retrovirus Research Center, Department of Medicine, Universidad Central del Caribe School of Medicine, Bayamon, Puerto Rico; 22 Department of Medicine, University of Washington School of Medicine, Seattle; 23 Department of Epidemiology, Biostatistics, and Occupational Health, McGill University, Montreal, Quebec, Canada; 24 Division of Infectious Diseases and Chronic Viral Illness Service, McGill University Health Centre, Montreal, Quebec, Canada

## Abstract

**Importance:**

People with HIV (PWH) are often coinfected with hepatitis B virus (HBV) and/or hepatitis C virus (HCV), leading to increased risk of developing hepatocellular carcinoma (HCC), but few cohort studies have had sufficient power to describe the trends of HCC incidence and risk among PWH in the combination antiretroviral therapy (cART) era.

**Objective:**

To determine the temporal trends of HCC incidence rates (IRs) and to compare rates by risk factors among PWH in the cART era.

**Design, Setting, and Participants:**

This cohort study used data from the North American AIDS Cohort Collaboration on Research and Design (NA-ACCORD) study, which was conducted between 1996 and 2015. NA-ACCORD pooled individual-level data from 22 HIV clinical and interval cohorts of PWH in the US and Canada. PWH aged 18 years or older with available CD4 cell counts and HIV RNA data were enrolled. Data analyses were completed in March 2020.

**Exposures:**

HBV infection was defined as detection of either HBV surface antigen, HBV e antigen, or HBV DNA in serum or plasma any time during observation. HCV infection was defined by detection of anti-HCV seropositivity, HCV RNA, or detectable genotype in serum or plasma at any time under observation.

**Main Outcomes and Measures:**

HCC diagnoses were identified on the basis of review of medical records or cancer registry linkage.

**Results:**

Of 109 283 PWH with 723 441 person-years of follow-up, the median (interquartile range) age at baseline was 43 (36-51) years, 93 017 (85.1%) were male, 44 752 (40.9%) were White, 44 322 (40.6%) were Black, 21 343 (19.5%) had HCV coinfection, 6348 (5.8%) had HBV coinfection, and 2082 (1.9%) had triple infection; 451 individuals received a diagnosis of HCC by 2015. Between the early (1996-2000) and modern (2006-2015) cART eras, the crude HCC IR increased from 0.28 to 0.75 case per 1000 person-years. HCC IRs remained constant among HIV-monoinfected persons or those coinfected with HBV, but from 1996 to 2015, IRs increased among PWH coinfected with HCV (from 0.34 cases/1000 person-years in 1996 to 2.39 cases/1000 person-years in 2015) or those with triple infection (from 0.65 cases/1000 person-years in 1996 to 4.49 cases/1000 person-years in 2015). Recent HIV RNA levels greater than or equal to 500 copies/mL (IR ratio, 1.8; 95% CI, 1.4-2.4) and CD4 cell counts less than or equal to 500 cells/μL (IR ratio, 1.3; 95% CI, 1.0-1.6) were associated with higher HCC risk in the modern cART era. People who injected drugs had higher HCC risk compared with men who had sex with men (IR ratio, 2.0; 95% CI, 1.3-2.9), adjusted for HBV-HCV coinfection.

**Conclusions and Relevance:**

HCC rates among PWH increased significantly over time from 1996 to 2015. PWH coinfected with viral hepatitis, those with higher HIV RNA levels or lower CD4 cell counts, and those who inject drugs had higher HCC risk.

## Key Points

**Question:**

Has the incidence rate or risk for liver cancer among people with HIV increased with the widespread availability of combination antiretroviral therapy?

**Findings:**

In this cohort study of 109 283 people with HIV in North America, the incidence rate of liver cancer increased between the early and modern combination antiretroviral therapy eras. People with HIV coinfected with hepatitis B virus and/or hepatitis C virus, those with higher HIV RNA levels and lower CD4 cell counts, and persons who inject drugs had a higher liver cancer risk.

**Meaning:**

These findings underscore the importance of achieving immune recovery along with monitoring for and long-term management of viral hepatitis among people with HIV.

## Teaser

This cohort study examines the trends of hepatocellular carcinoma (HCC) incidence rates and compares rates by risk factors among people in the US and Canada with HIV infection in the combination antiretroviral therapy era.

## Introduction

Globally, hepatocellular carcinoma (HCC) incidence increased by 75% between 1990 and 2015, making HCC one of the most common cancers and leading causes of cancer death worldwide.^1^ North America is projected to have among the largest increases in HCC incidence rates (IRs) by 2030.^2^ Consistent with global objectives for eliminating viral hepatitis, reduction in the incidence and mortality of HCC represents an immediate public health challenge.^3^

People with HIV (PWH) have a higher burden of HCC^4,5^ and end-stage liver disease^6,7,8^ compared with people without HIV because of the high prevalence of coinfection with hepatitis B virus (HBV) and/or hepatitis C virus (HCV),^9,10,11^ risk behaviors,^12^ and immune dysfunction.^13,14^ Since the advent of combination antiretroviral therapy (cART), PWHs have prolonged survival, resulting in a notable increase in the number of PWHs older than 50 years,^15^ concurrent with the age when many cancers are diagnosed in the general population.^16^ Previous studies^4,17,18,19,20^ found increasing incidence of several non–AIDS-defining cancers, including HCC, in the modern cART era, in part attributable to aging of the PWH population.

Understanding incidence trends and risk factors associated with HCC in aging PWH is critical to developing evidence-based interventions aimed at reducing HCC burden. Furthermore, trends and risk factors associated with HCC in North America, which had progressive cART uptake before the widespread use of hepatitis C treatments, may inform expectations for other regions with a substantial burden of HIV and HBV-HCV coinfection but with delayed cART scale-up and limited access to viral hepatitis treatment. However, most previous studies had limited HCC cases and were underpowered to evaluate this trend among PWH. We assessed temporal trends for HCC among PWH in the cART era, comparing HCC rates by viral hepatitis infection, risk populations, and HIV disease severity using data from a large cohort consortium, the North American AIDS Cohort Collaboration on Research and Design (NA-ACCORD).

## Methods

### Study Design and Population

NA-ACCORD is a collaborative study that pools individual-level data from 22 HIV clinical and interval cohorts in Canada and US. Design, data collection, and data harmonization methods have been previously described.^21,22^ Individuals were enrolled into NA-ACCORD from contributing cohorts when they had 2 or more clinical or study visits within 12 months. Each cohort reported demographic, medication, laboratory, diagnoses, and vital status data annually to the Data Management Core for data harmonization and quality control. Data were then sent to the Epidemiology/Biostatistics Core, where analytic-ready summary files were completed. Institutional review boards at each study location approved the study protocol, and each participant provided written informed consent. Consortium activities were approved by the Johns Hopkins University institutional review board. This study followed the Strengthening the Reporting of Observational Studies in Epidemiology (STROBE) reporting guideline.

We included PWH aged 18 years or older with available CD4 cell counts and HIV RNA data between 1996 and 2015; 11.6% of participants were excluded because of missing data (inclusion criteria are shown in eFigure 1 in the Supplement). Three cART eras were analyzed: early cART (1996-2000), middle cART (2001-2005), and modern cART (2006-2015), corresponding to temporal changes in cART regimens.^22,23,24^ Person-time at risk for HCC accrued from the later of the first date of the cART era, participants’ enrollment into NA-ACCORD, first available CD4 cell count and/or HIV RNA level measurement, or beginning of cohort-specific cancer diagnosis ascertainment. Participants no longer contributed person-time at the first date of HCC diagnosis, death, December 31, 2015, or end of cohort-specific cancer ascertainment.

### HCC Case Definition

HCC diagnoses (451 cases) were collected by extraction from medical records using a Web-based standardized abstraction protocol for 415 cases; 36 cases were identified by linkage to cancer registries.^4^ Among non–cancer registry cohorts, all reported cases were verified by trained medical record abstracters under the supervision of physicians. Histopathology, laboratory, and imaging results were used to adjudicate all cases. The date of first confirmed HCC diagnosis was used for analysis.

### Primary Exposure: HBV and HCV Infection

HBV infection was defined by the detection of either hepatitis B surface antigen, hepatitis B e antigen, or HBV DNA in blood during observation. HCV infection was defined by the detection of anti-HCV seropositivity, HCV RNA, or detectable genotype in blood during observation. HBV or HCV infection status was time-varying and remained fixed after positive status was defined. Individuals who had not been classified as having either HBV or HCV coinfection during follow-up were considered to have HIV monoinfection. Four groups were defined: HIV monoinfection (HBV and HCV negative), HIV-HBV coinfection, HIV-HCV coinfection, and triple infection (HIV, HBV, and HCV positive).

### Other Covariates

Sociodemographic information (age, sex, and race) and HIV transmission risk (men who have sex with men [MSM], people who inject drugs [PWID], heterosexual contact, and other or unknown) were collected through self-report or medical record review at enrollment. Individuals who were both MSM and PWID (1229 individuals [1.1%]) were categorized as PWID. Antiretroviral regimens were characterized at beginning of each cART era. CD4 cell counts (cells per microliter) were measured at the time closest to entry of each cART era or beginning of cancer observation within that period. HIV RNA levels (copies per milliliter) were measured 2 years (18-30 months) before HCC diagnosis or censoring (loss-to-follow-up or end of observation). FIB-4 (fibrosis-4) scores were calculated using standard methods based on studies conducted in patients with chronic hepatitis, with a value greater than 3.25 considered advanced liver fibrosis.^25^

### Statistical Analysis

The χ^2^ or Wilcoxon test (2-sided) was used to compare differences in baseline characteristics by subsequent diagnosis of HCC. Significance was set at *P* < .05. Poisson regression models were used to estimate HCC IRs per 1000 person-years. In case of overdispersion in models, generalized estimating equation models were used as an alternative. We compared IR ratios (IRRs) with 95% CIs by viral hepatitis coinfection groups, transmission risk groups, CD4 cell counts (≤500 vs >500 cells/μL), and HIV RNA levels (<500 vs ≥500 copies/mL). IRRs were adjusted for age group (<40, 40-49, 50-59, and ≥60 years), sex, race (White, Black, and other or unknown), and cohort; additional adjustment for viral hepatitis coinfection was performed when appropriate. Age-specific cumulative incidence of HCC by viral hepatitis coinfection group was calculated. Sensitivity analysis compared models with and without accounting for competing risks of death. Statistical analyses were conducted using SAS statistical software version 9.4 (SAS Institute) and completed in March 2020.

## Results

### Characteristics of Participants

A total of 109 283 participants with 723 441 person-years of follow-up (median [interquartile range {IQR}], 5 [2-10] years) from 22 cohorts were included. At baseline, the median (IQR) age was 43 (36-51) years; 93 017 participants (85.1%) were male, 44 752 (40.9%) were White, and 44 322 (40.6%) were Black. Overall, 21 343 participants (19.5%) had HCV coinfection, 6348 (5.8%) had HBV coinfection, and 2082 (1.9%) had triple infection. Participants with HCC were almost uniformly male (437 men [96.9%]) even though the study population was 85.1% male. Persons with FIB-4 scores greater than 3.25 at baseline were more likely than those with lower scores to later receive a diagnosis of HCC (Table 1).

**Table 1.** Baseline Characteristics of Study Participants by HCC Status

Baseline characteristics	Patients, No. (%)			*P* value^a^
	Overall (N = 109 283)	Non-HCC (n = 108 832)	HCC (n = 451)	
Follow-up time, median (IQR), y	5 (2-10)	5 (2-11)	7 (3-10)	<.001
Country				
US	102 134 (93.5)	101 691 (93.4)	443 (98.2)	<.001
Canada	7149 (6.5)	7141 (6.6)	8 (1.8)	
Age, median (IQR), y	43 (36-51)	43 (36-51)	51 (46-56)	<.001
<40	42 096 (38.5)	42 068 (38.7)	28 (6.2)	
40-49	37 321 (34.2)	37 144 (34.1)	177 (39.2)	
50-59	22 069 (20.2)	21 883 (20.1)	186 (41.2)	
≥60	7797 (7.1)	7737 (7.1)	60 (13.4)	
Male	93 017 (85.1)	92 580 (85.1)	437 (96.9)	<.001
Race				
White	44 752 (40.9)	44 592 (41.0)	160 (35.5)	.002
Black	44 322 (40.6)	44 102 (40.5)	220 (48.8)	
Other or unknown	20 209 (18.5)	20 138 (18.5)	71 (15.7)	
HIV transmission risk				
MSM	35 465 (32.4)	35 410 (32.5)	55 (12.2)	<.001
PWID	20 648 (18.9)	20 437 (18.8)	211 (46.8)	
Heterosexual	16 833 (15.4)	16 820 (15.5)	13 (2.9)	
Other or unknown	36 337 (33.3)	36 165 (33.2)	172 (38.1)	
Viral hepatitis status^b^				
HIV monoinfected	76 897 (70.4)	76 860 (70.6)	37 (8.2)	<.001
HIV and HBV	6348 (5.8)	6269 (5.8)	79 (17.5)	
HIV and HCV	21 343 (19.5)	21 057 (19.3)	286 (63.4)	
HIV, HBV, and HCV	2082 (1.9)	2041 (1.9)	41 (9.1)	
Unassessed	2613 (2.4)	2605 (2.4)	8 (1.8)	
Antiretroviral therapy use at baseline	59 250 (54.2)	58 958 (54.2)	292 (64.7)	<.001
CD4 cell count, cells/μL				
<200	29 981 (27.4)	29 871 (27.4)	110 (24.4)	.02
200-350	23 144 (21.2)	23 024 (21.2)	120 (26.6)	
351-500	21 400 (19.6)	21 306 (19.6)	94 (20.9)	
>500	33 901 (31.0)	33 775 (31.0)	126 (27.9)	
Missing	857 (0.8)	856 (0.8)	1 (0.2)	
HIV RNA level, copies/mL				
<500	39 794 (36.4)	39 580 (36.4)	214 (47.5)	<.001
≥500	65 994 (60.4)	65 763 (60.4)	231 (51.2)	
Missing	3495 (3.2)	3489 (3.2)	6 (1.3)	
Alcohol use				
Never	20 417 (18.7)	20 373 (18.7)	44 (9.8)	.87
Ever	10 172 (9.3)	10 151 (9.3)	21 (4.7)	
Missing	78 694 (72.0)	78 308 (72.0)	386 (85.6)	
Smoking				
Never	15 861 (14.5)	15 839 (14.5)	22 (4.9)	.02
Ever	44 366 (40.6)	44 261 (40.7)	105 (23.3)	
Missing	49 056 (44.9)	48 732 (44.8)	324 (71.8)	
Fibrosis-4 score				
<1.45	51 642 (47.3)	51 595 (47.4)	47 (10.4)	<.001
1.45-3.25	19 865 (18.2)	19 723 (18.1)	142 (31.5)	
>3.25	6164 (5.6)	6030 (5.5)	134 (29.7)	
Missing	31 612 (28.9)	31 484 (28.9)	128 (28.4)	

Abbreviations: HBV, hepatitis B virus; HCC, hepatocellular carcinoma; HCV, hepatitis C virus; IQR, interquartile range; MSM, men who have sex with men; PWID, people who injected drugs.

^a^ *P* values were calculated for comparison of HCC vs non-HCC using χ^2^ test for categorical variables and the Wilcoxon rank sum tests for continuous variables.

^b^ HBV infection was defined by detection of either hepatitis B surface antigen, hepatitis B e antigen, or HBV DNA in serum or plasma at any time during observation. HCV infection was defined by the detection of HCV antibody seropositivity, HCV RNA, or detectable genotype in serum or plasma at any time under observation.

### Incidence of HCC

A total of 451 HCC cases (overall IR, 0.62 case/1000 person-years; 95% CI, 0.57-0.68 case/1000 person-years) were observed. The crude IR of HCC increased from 0.28 to 0.75 case per 1000 person-years between the early and modern cART eras (eFigure 2 in the Supplement). After adjusting for covariates, there was a 72% increase in HCC IR (adjusted IRR, 1.72; 95% CI, 1.03-2.87) (eTable 1 in the Supplement). Age-specific IRs of HCC increased by calendar period only among those aged 50 years or older (eTable 2 in the Supplement). Cumulative incidence of HCC accounting for competing risks of death showed little difference compared with primary models (eFigure 3 in the Supplement).

### IRs, IRRs, and Cumulative Incidence of HCC by HBV-HCV Coinfection

**Figure 1.**
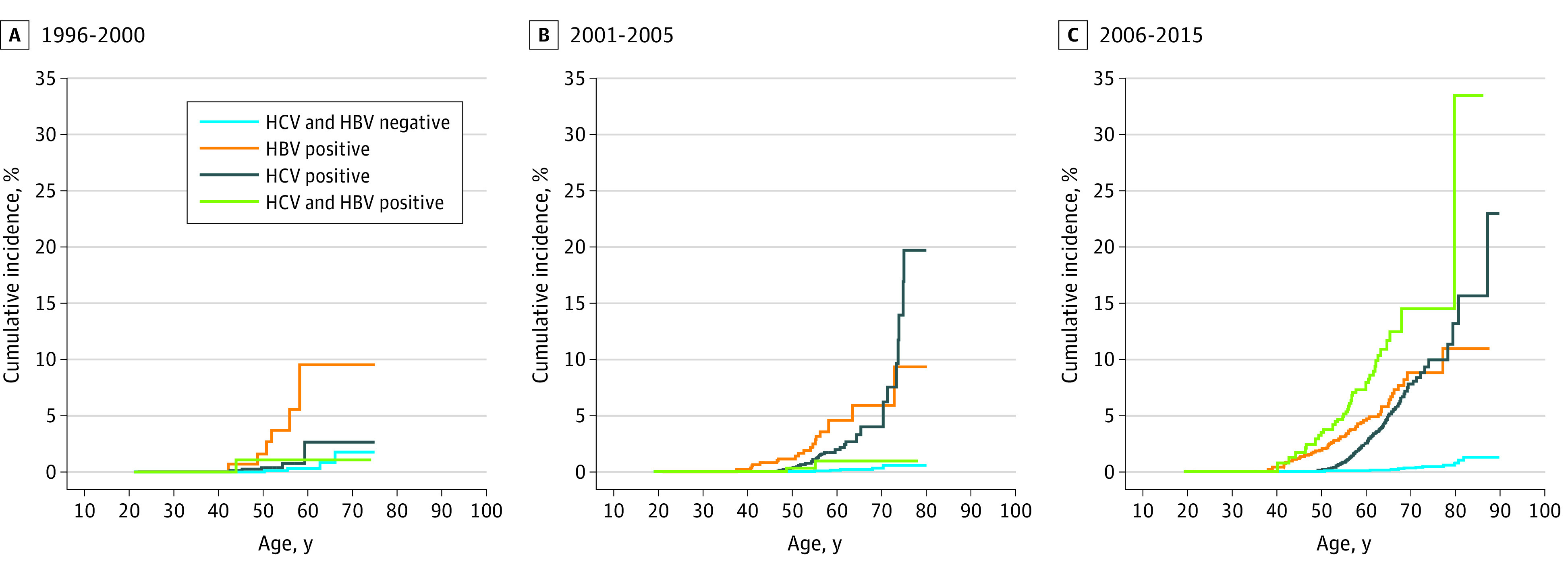
Cumulative Incidence of Hepatocellular Carcinoma by Age and Viral Hepatitis Coinfection Groups Stratified by Calendar Era Hepatitis B virus (HBV) infection was defined by detection of either hepatitis B surface antigen, hepatitis B e antigen, or HBV DNA in serum or plasma at any time during observation. Hepatitis C virus (HCV) infection was defined by the detection of HCV antibody seropositivity, HCV RNA, or detectable genotype in serum or plasma at any time under observation.

There was no substantial change in crude IRs of HCC across 3 eras among the HIV monoinfection group (eTable 3 in the Supplement). Rates of HCC among PWH coinfected with HBV were elevated in the early cART era and then increased in the modern cART era, whereas rates substantially increased across 3 eras among PWH coinfected with HCV (early cART: IR, 0.34 case/1000 person-years [95% CI, 0.14-0.82 case/1000 person-years]; middle cART: IR, 1.09 cases/1000 person-years [95% CI, 0.84-1.42 cases/1000 person-years]; modern cART: IR, 2.39 cases/1000 person-years; 95% CI, 2.10-2.73 cases/1000 person-years) or those with triple infection (early cART: IR, 0.65 case/1000 person-years [95% CI, 0.09-4.6 cases/1000 person-years]; middle cART: IR, 0.36 case/1000 person-years [95% CI, 0.09-1.44 cases/1000 person-years]; modern cART: IR, 4.49 cases/1000 person-years [95% CI, 3.27-6.18 cases/1000 person-years]) (Table 2 and eTable 3 in the Supplement). HBV or HCV coinfection was associated with a more than 20 times higher incidence, and triple infection was associated with nearly 40 times higher incidence of HCC compared with HIV monoinfection in the modern cART era. Coinfection with HBV and/or HCV was also associated with higher age-specific cumulative incidence of HCC compared with HIV monoinfection (Figure 1). Results from sensitivity analysis in a subset of the cohort (35 189 participants [32.2%]) further adjusted for alcohol and smoking consumption were consistent with primary models (eTable 4 in the Supplement).

**Table 2.** IR and IRR of HCC by Viral Hepatitis Coinfection Groups and Calendar Period

cART era and viral hepatitis coinfection groups^a^	Patients, No.	HCC cases, No.	IR, HCC cases/1000 person-years	IRR (95% CI)	
				Crude	Adjusted^b^
Early cART era (1996-2000)					
Overall	33 666	16	0.28 (0.17-0.46)	NA	NA
HIV monoinfection^c^	21 641	4	0.11 (0.04-0.28)	1 [Reference]	1 [Reference]
HIV and HCV^d^	9029	5	0.34 (0.14-0.82)	3.2 (0.9-12.0)	2.3 (0.6-9.1)
HIV and HBV^e^	2118	6	1.66 (0.74-3.69)	15.6 (4.4-55.2)	15.4 (4.3-55.5)
HIV, HBV, and HCV^f^	878	1	0.65 (0.09-4.6)	6.1 (0.7-54.4)	4.9 (0.5-45.8)
Middle cART era (2001-2005)					
Overall	62 600	87	0.43 (0.35-0.53)	NA	NA
HIV monoinfection^c^	40 315	7	0.05 (0.03-0.11)	1 [Reference]	1 [Reference]
HIV and HCV^d^	14 598	55	1.09 (0.84-1.42)	20.0 (9.1-43.8)	18.2 (8.1-40.4)
HIV and HBV^e^	4177	19	1.36 (0.87-2.14)	24.9 (10.5-59.2)	25.4 (10.7-60.7)
HIV, HBV, and HCV^f^	1567	2	0.36 (0.09-1.44)	6.6 (1.4-31.8)	6.6 (1.4-32.1)
Modern cART era (2006-2015)					
Overall	83 124	348	0.75 (0.68-0.84)	NA	NA
HIV monoinfection^c^	62 354	26	0.08 (0.05-0.12)	1 [Reference]	1 [Reference]
HIV and HCV^d^	16 547	226	2.39 (2.10-2.73)	29.7 (19.8-44.6)	23.8 (15.8-35.9)
HIV and HBV^e^	4628	54	2.09 (1.60-2.73)	25.9 (16.2-41.3)	23.7 (14.9-37.9)
HIV, HBV, and HCV^f^	1442	38	4.49 (3.27-6.18)	55.7 (33.8-91.8)	45.7 (27.7-75.5)

Abbreviations: cART, combination antiretroviral therapy; HBV, hepatitis B virus; HCC, hepatocellular carcinoma; HCV, hepatitis C virus; IR, incidence rate; IRR, incidence rate ratio; NA, not applicable.

^a^ HBV infection was defined by detection of either hepatitis B surface antigen, hepatitis B e antigen, or HBV DNA in serum or plasma at any time during observation. HCV infection was defined by the detection of HCV antibody seropositivity, HCV RNA, or detectable genotype in serum or plasma at any time under observation.

^b^ IRRs were adjusted for age group (<40, 40-49, 50-59, ≥60 years), sex, race, and cohort.

^c^ HIV monoinfection was defined as HIV positive but HBV negative and HCV negative.

^d^ HCV-positive only was defined as HCV positive and HBV negative or not assessed.

^e^ HBV-positive only was defined as HBV positive and HCV negative or not assessed.

^f^ Denotes positivity for HIV, HBV, and HCV.

**Figure 2.**
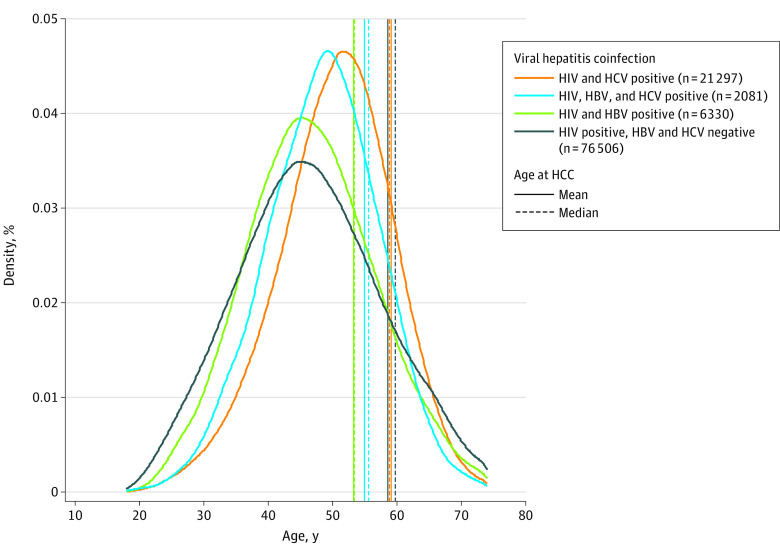
Age Distribution and Age of Hepatocellular Carcinoma (HCC) Onset by Viral Hepatitis Coinfection Groups Hepatitis B virus (HBV) infection was defined by detection of either hepatitis B surface antigen, hepatitis B e antigen, or HBV DNA in serum or plasma at any time during observation. Hepatitis C virus (HCV) infection was defined by the detection of HCV antibody seropositivity, HCV RNA, or detectable genotype in serum or plasma at any time under observation. Dashed vertical lines denote median age at HCC diagnosis in each group, and solid vertical lines denote mean age at HCC diagnosis.

We compared age at HCC diagnosis with the underlying age distribution of each coinfection group (Figure 2 and eTable 5 in the Supplement) and observed that the HIV monoinfected group had the oldest age at HCC diagnosis and the greatest difference from the median age of the underlying population. Despite similar underlying age distributions (Figure 2), persons coinfected with HIV and HBV were markedly younger at HCC diagnosis (median [IQR], 54 [47-58] years) than HIV monoinfected persons (median [IQR], 61 [53-68] years).

### IRs of HCC by HIV Disease Severity

In the modern cART era (Table 3), PWH with recent HIV RNA levels greater than or equal to 500 copies/mL had an 80% increased HCC IR (IRR, 1.8; 95% CI, 1.4-2.4) compared with those with HIV RNA levels less than 500 copies/mL. Similarly, PWH with lower CD4 cell counts (≤500 cell/μL) had 30% increased HCC IR vs PWH with higher CD4 cell counts (IR 1.3; 95% CI, 1.0-1.6). Both outcomes were independent of viral hepatitis coinfection and covariates. Higher HCC rates associated with more advanced HIV disease severity were not observed in earlier calendar periods (eTables 6, eTable 7, and eTable 8 in the Supplement).

**Table 3.** IR and IRR of HCC by CD4 Cell Counts, CD4 Cell Percentage, and HIV Viral Load in the Modern Combination Antiretroviral Therapy Era

HIV categories^a^	Patients, No.	HCC events, No.	Person-years, No.	IR, HCC cases/1000 person-years	IRR (95% CI)	
					Unadjusted	Adjusted^b^
HIV RNA viral load, copies/mL						
<500	56 668	219	353 162	0.62 (0.54-0.71)	1 [Reference]	1 [Reference]
≥500	17 569	75	68 491	1.1 (0.87-1.37)	1.8 (1.4-2.3)	1.8 (1.4-2.4)
CD4 cell counts, cells/μL^a^						
≤500	48 997	223	258 035	0.86 (0.76-0.99)	1.4 (1.1-1.8)	1.3 (1.0-1.6)
>500	29 407	96	159 457	0.60 (0.49-0.74)	1 [Reference]	1 [Reference]
CD4 cell percentage^a^						
≤29%	52 315	225	282 004	0.8 (0.7-0.91)	1.1 (0.9-1.4)	0.9 (0.7-1.2)
>29%	24 200	93	128 227	0.73 (0.59-0.89)	1 [Reference]	1 [Reference]

Abbreviations: HCC, hepatocellular carcinoma; IR, incidence rate; IRR, incidence rate ratio.

^a^ CD4 cell count and CD4 cell percentage were assessed at the entry of each era as CD4 closest to the entry within window of 6 months prior to 3 months after the entry date; HIV viral load was assessed at 30 to 18 months prior to HCC diagnosed or time of censoring of the calendar period.

^b^ IRRs were adjusted for age (<40, 40-49, 50-59, ≥60 years), sex, race, cohort, and viral hepatitis infection.

### HCC Rates by HIV Transmission Group

PWID had higher HCC incidence and increased IRRs compared with MSM (eTable 9 in the Supplement). We hypothesized that this was largely associated with differences in viral hepatitis coinfection. eFigure 4 in the Supplement demonstrates the distribution of known HIV transmission risk groups by viral hepatitis status. PWID represented most of the HCV coinfection group, whereas MSM represented most of the HBV coinfection group.

In models adjusting for demographic characteristics and cohort, PWID had higher HCC IRs than MSM (eTable 9 in the Supplement), and the IR was increased significantly in the modern cART era (IRR, 2.7; 95% CI, 1.88-3.86) (eTable 10 in the Supplement). Increased HCC risk for PWID was attenuated but not eliminated after controlling for viral hepatitis coinfection (IRR, 2.0; 95% CI, 1.3-2.9) (model B in eTable 9 in the Supplement).

## Discussion

We examined HCC temporal trends and risk factors among PWH in North America over a 2-decade period, representing one of the largest and longest prospective studies with validated cancer outcomes of HCC in the context of HIV. HCC is among the most lethal cancers worldwide and is one of the few malignant entities with an increasing incidence^26^ and mortality in the US in recent decades.^27^ PWH had a more than 3 times higher IR for HCC compared with the US general population rates observed in the Surveillance, Epidemiology, and End Results-Medicare linkage (0.75 vs 0.23 case/1000 person-years),^26^ demonstrating the increased relative burden of HCC among PWH. Similar to other studies conducted among PWH,^28,29,30^ our study confirmed that the IR of HCC is increasing in North America. The increase in both IR and number of cases of HCC was most noticeable among individuals aged 50 years or older, which suggests that population age structure change and survival benefits are the major contributing factor for the increased IR. The use of cART has prolonged survival among PWH, which suggests the burden of HCC might further increase as the population continues to age with longer exposure to both HIV and chronic liver diseases. When comparing the modern vs the early cART eras, the IRRs of HCC remained significant even after controlling for age groups, suggesting that additional factors could be associated with the increased incidence. The increased rate might also be attributed to other nonviral causes (eg, alcohol abuse, obesity, diabetes, and nonalcoholic fatty liver diseases). In the general population, the prevalence of alcohol abuse and nonalcoholic fatty liver diseases has been increasing in the US concurrent with our study period.^31,32^ These nonviral risk factors could act synergistically with viral hepatitis and might play a role in the increase.

PWH coinfected with HBV and/or HCV had substantially higher HCC rates and had earlier HCC diagnoses compared with HIV monoinfected persons, and this difference was most pronounced in the modern cART era. These findings are consistent with the previous observation that viral hepatitis coinfection is associated with a higher risk of end-stage liver disease.^33^ The incidence of HCC among PWH coinfected with HCV was higher in our study than that observed in the study by Gjærde et al^30^ (2.39 vs 1.3 cases/1000 person-years), but lower than that in the study by Kramer et al^34^ (2.39 vs 4.44 cases/1000 person-years). Data in previous literature on the incidence of HCC among PWH coinfected with HBV remain sparse. HCC rates among PWH coinfected with HBV and/or HCV also increased substantially in the middle and modern cART eras. The increased trend in HCV and HBV-related HCC among PWH in the last 2 cART eras is comparable to that observed in the US population,^26^ despite a notably younger age distribution in NA-ACCORD. Furthermore, rates of HCC are substantially higher among PWH compared with the US general population (approximately 25 times higher among PWH coinfected with HCV and more than 100 times higher among PWH coinfected with HBV).^26^ PWH with viral hepatitis coinfection have elevated HCC incidence rates that are approaching the level suggested to benefit from HCC surveillance in other selected populations.^35^ Data from the current study warrant further investigation, including randomized clinical trials to evaluate the efficacy of more active cancer surveillance among PWH.

As many as 25% of PWH are coinfected with chronic HCV^36,37^ and 8% to 15% of PWH are coinfected with chronic HBV^9^ globally because of shared routes of transmission.^37^ The substantial contributions of chronic HBV and HCV infection to prolonged liver inflammation, regeneration, fibrosis, and ultimately HCC have been well described elsewhere.^38,39^ Since the introduction of highly effective direct-acting antivirals (DAAs), the landscape of hepatitis C care has dramatically changed.^40,41^ However, barriers to treatment uptake, including education, screening, and high cost, have prevented some PWH coinfected with HCV from benefiting from DAAs.^42,43^ Appreciating the substantial risk associated with HIV-HCV coinfection and the public health burden, access to hepatitis C treatment among PWH should be prioritized. Conversely, although lacking a cure, antiviral therapy has been shown to provide long-term benefits for patients with chronic HBV without HIV.^44,45^ Several ART medications (eg, lamivudine, emtricitabine, tenofovir, and emtricitabine) are also active against HBV. Through effectively controlling both HBV and HIV viral replication, these therapies could improve liver-related outcomes.^8,46^ However, despite the widespread use of HBV-active ART, we previously reported that a substantial number of PWH with HBV were not receiving appropriate regimens.^33^ Given the substantial prevalence of HBV coinfection and increased risk of HCC, vaccination against HBV and/or testing for protective antibodies remains vital among PWH. Broader uptake and early initiation of HBV-active ART among PWH coinfected with HBV also remains a critical secondary prevention strategy.^47,48,49^

Previous studies^50,51^ suggested that a longer duration of HIV viremia and lower CD4 cell counts (<200 cells/μL) were associated with higher risk of HCC. Our findings suggest that even recent exposure to higher viral load and to less pronounced immunosuppression (CD4 cell counts ≤500 cell/μL) could also be associated with higher HCC risk. PWH suffer from persistent immune dysregulation and chronic inflammation,^14^ factors that could further intensify liver fibrosis progression and HCC development among PWH with chronic HBV or HCV infection.^52^ Although lower CD4 cell count could be a reflection of lymphopenia associated with advanced liver diseases resulting from chronic hepatitis (eg, portal hypertension and cirrhosis) instead of immune dysfunction,^53^ our findings suggest that the association was independent of viral hepatitis coinfection. Effective control of HIV replication is critical to controlling hepatic inflammation and fibrosis among PWH with or without viral hepatitis coinfection.^54,55^

Because of the nature of transmission of HCV and HBV, PWID and MSM represent a large proportion of those with HCV or HBV coinfection, respectively. PWID had a higher HCC risk compared with MSM, most notably in the modern cART era. However, viral hepatitis coinfection does not appear to fully explain the risk difference between PWID and MSM. Other risk behaviors (eg, alcohol consumption and smoking) and potential misclassification might explain such disparities. Because PWID more commonly have lower socioeconomic status, limited access to care, and ongoing injection drug use,^40,42^ it is vital to address other factors and health disparities to ameliorate the overall burden of HCC among aging PWH. Treatment strategies focused on HCV screening and early intervention for chronic HCV or HBV among PWID, as well as long-term management of risk behaviors, should be further explored.

### Limitations

This study has limitations that should be addressed. First, although NA-ACCORD is representative of patients with HIV successfully linked to health care in North America, we were not able to make inferences about PWH who are not receiving health care. Second, although our observation time and sample size are large for an HCC study, granular data on potential risk factors (eg, natural clearance vs treated vs chronic HCV, alcohol, smoking, or obesity) were not available across all cohorts, making investigation of other important etiologic factors a limitation. However, results from a sensitivity analysis within a subset (32.2% of the cohort) with data on alcohol and smoking were consistent with our primary observations. Third, not all participants had been tested for HBV and HCV in NA-ACCORD, and testing for HBV and/or HCV in clinical cohorts was based on clinical decision. We might have misclassified some HCV- or HBV-coinfected patients in the HIV monoinfection group and underestimated the risk of HCC in the HBV and/or HCV coinfection groups. Fourth, completeness of HCC case ascertainment has been estimated to be robust at individual sites but not uniform across all sites, where sites could use either the cancer registry or electronic medical record ascertainment or both methods for case definition. However, the NA-ACCORD Data Management Core has conducted quality assessment and control of case definitions to minimize any differences through the harmonization process.^21,22^ Fifth, DAAs uptake for hepatitis C treatment has been limited in the current database and rate of interferon-based regimens were low in NA-ACCORD before the DAA era. Therefore, we were not able to draw further implications for HCC risk among PWH receiving DAAs treatment. With further data accumulation in NA-ACCORD, this will be a focus of future analyses.

## Conclusions

In conclusion, we used a large HIV collaborative study design to determine rates for HCC among PWH in North America in the 20 years since the availability of cART. The increases in HCC rates are of concern and may lead to continuing increases in HCC-related mortality in coming years. PWH coinfected with viral hepatitis with higher HIV RNA levels or lower CD4 cell counts or who contracted HIV through injection drug use had higher HCC rates. These findings underscore the importance of achieving immune recovery and monitoring for and long-term management of viral hepatitis among PWH.

## Notes

Supplement. **eFigure 1.** Flow Diagram for Sample Selection **eFigure 2.** Crude Incidence Rates of Hepatocellular Carcinoma in NA-ACCORD by cART Eras, 1996-2015 **eFigure 3.** Crude Cumulative Incidence Function (CIF) of HCC Compared to CIF Incorporating Competing Risks of Deaths Using Fine & Gray Methods, by Calendar Periods **eFigure 4. **Distribution of HIV Transmission Risk Group by Viral Hepatitis Coinfection Status in NA-ACCORD **eTable 1. **HCC Incidence Rates and Incidence Rate Ratios in NA-ACCORD, by Calendar Period **eTable 2. **Age Specific HCC Incidence in NA-ACCORD by Calendar Periods **eTable 3. **Hepatocellular Carcinoma Incidence Rates and Incidence Rate Ratios in NA-ACCORD Before and After 2006 by Viral Hepatitis Coinfection Groups **eTable 4. **Sensitivity Analysis of Hepatocellular Carcinoma Incidence Rates by Hepatitis C and Hepatitis B and ART Era Controlled for Alcohol and Smoking Behavior **eTable 5. **Distribution of Age at Hepatocellular Carcinoma Diagnosis and Age of Underlying Population by Viral Hepatitis Coinfection **eTable 6. **Incidence Rates and Incidence Rate Ratios of Hepatocellular Carcinoma by CD4 Counts by cART Periods **eTable 7. **Incidence Rates and Incidence Rate Ratios of Hepatocellular Carcinoma by CD4 Percentage by cART **eTable 8. **Incidence Rates and Incidence Rate Ratios of Hepatocellular Carcinoma by HIV Viral Load by cART Periods **eTable 9. **Incidence Rates and Incidence Rate Ratios of Hepatocellular Carcinoma by HIV Transmission Risk Group and Calendar Years **eTable 10. **Hepatocellular Carcinoma Incidence Rates and Incidence Rate Ratios Before and After 2006 by HIV Transmission Risk Groups
