# Supplementary material for: Trends in Hepatocellular Carcinoma Incidence and Risk Among Persons With HIV in the US and Canada, 1996-2015
Source: JAMA Netw Open. 2021 Feb 17;4(2):e2037512. doi: 10.1001/jamanetworkopen.2020.37512 (PMC7890526; doi:10.1001/jamanetworkopen.2020.37512)
Supplement: Supplement. — eFigure 1. Flow Diagram for Sample Selection eFigure 2. Crude Incidence Rates of Hepatocellular Carcinoma in NA-ACCORD by cART Eras, 1996-2015 eFigure 3. Crude Cumulative Incidence Function (CIF) of HCC Compared to CIF Incorporating Competing Risks of Deaths Using Fine & Gray Methods, by Calendar Periods eFigure 4. Distribution of HIV Transmission Risk Group by Viral Hepatitis Coinfection Status in NA-ACCORD eTable 1. HCC Incidence Rates and Incidence Rate Ratios in NA-ACCORD, by Calendar Period eTable 2. Age Specific HCC Incidence in NA-ACCORD by Calendar Periods eTable 3. Hepatocellular Carcinoma Incidence Rates and Incidence Rate Ratios in NA-ACCORD Before and After 2006 by Viral Hepatitis Coinfection Groups eTable 4. Sensitivity Analysis of Hepatocellular Carcinoma Incidence Rates by Hepatitis C and Hepatitis B and ART Era Controlled for Alcohol and Smoking Behavior eTable 5. Distribution of Age at Hepatocellular Carcinoma Diagnosis and Age of Underlying Population by Viral Hepatitis Coinfection eTable 6. Incidence Rates and Incidence Rate Ratios of Hepatocellular Carcinoma by CD4 Counts by cART Periods eTable 7. Incidence Rates and Incidence Rate Ratios of Hepatocellular Carcinoma by CD4 Percentage by cART eTable 8. Incidence Rates and Incidence Rate Ratios of Hepatocellular Carcinoma by HIV Viral Load by cART Periods eTable 9. Incidence Rates and Incidence Rate Ratios of Hepatocellular Carcinoma by HIV Transmission Risk Group and Calendar Years eTable 10. Hepatocellular Carcinoma Incidence Rates and Incidence Rate Ratios Before and After 2006 by HIV Transmission Risk Groups [file jamanetwopen-e2037512-s001.pdf]

Supplemental Online Content

Sun J, Althoff KN, Jing Y, et al; North American AIDS Cohort Collaboration on Research and Design of IeDEA. Trends in hepatocellular carcinoma incidence and risk among persons with HIV in the US and Canada, 1996-2015. *JAMA Netw Open*. 2021;4(2):e2037512. doi:10.1001/jamanetworkopen.2020.37512

eFigure 1. Flow Diagram for Sample Selection

eFigure 2. Crude Incidence Rates of Hepatocellular Carcinoma in NA-ACCORD by cART Eras, 1996-2015

eFigure 3. Crude Cumulative Incidence Function (CIF) of HCC Compared to CIF Incorporating Competing Risks of Deaths Using Fine & Gray Methods, by Calendar Periods

eFigure 4. Distribution of HIV Transmission Risk Group by Viral Hepatitis Coinfection Status in NA-ACCORD

eTable 1. HCC Incidence Rates and Incidence Rate Ratios in NA-ACCORD, by Calendar Period

eTable 2. Age Specific HCC Incidence in NA-ACCORD by Calendar Periods

eTable 3. Hepatocellular Carcinoma Incidence Rates and Incidence Rate Ratios in NA-ACCORD Before and After 2006 by Viral Hepatitis Coinfection Groups

eTable 4. Sensitivity Analysis of Hepatocellular Carcinoma Incidence Rates by Hepatitis C and Hepatitis B and ART Era Controlled for Alcohol and Smoking Behavior

eTable 5. Distribution of Age at Hepatocellular Carcinoma Diagnosis and Age of Underlying Population by Viral Hepatitis Coinfection

eTable 6. Incidence Rates and Incidence Rate Ratios of Hepatocellular Carcinoma by CD4 Counts by cART Periods

eTable 7. Incidence Rates and Incidence Rate Ratios of Hepatocellular Carcinoma by CD4 Percentage by cART

eTable 8. Incidence Rates and Incidence Rate Ratios of Hepatocellular Carcinoma by HIV Viral Load by cART Periods

eTable 9. Incidence Rates and Incidence Rate Ratios of Hepatocellular Carcinoma by HIV Transmission Risk Group and Calendar Years

eTable 10. Hepatocellular Carcinoma Incidence Rates and Incidence Rate Ratios Before and After 2006 by HIV Transmission Risk Groups

This supplemental material has been provided by the authors to give readers additional information about their work.

© 2021 Sun J et al. *JAMA Network Open.*

eFigure 1. Flow Diagram for Sample Selection

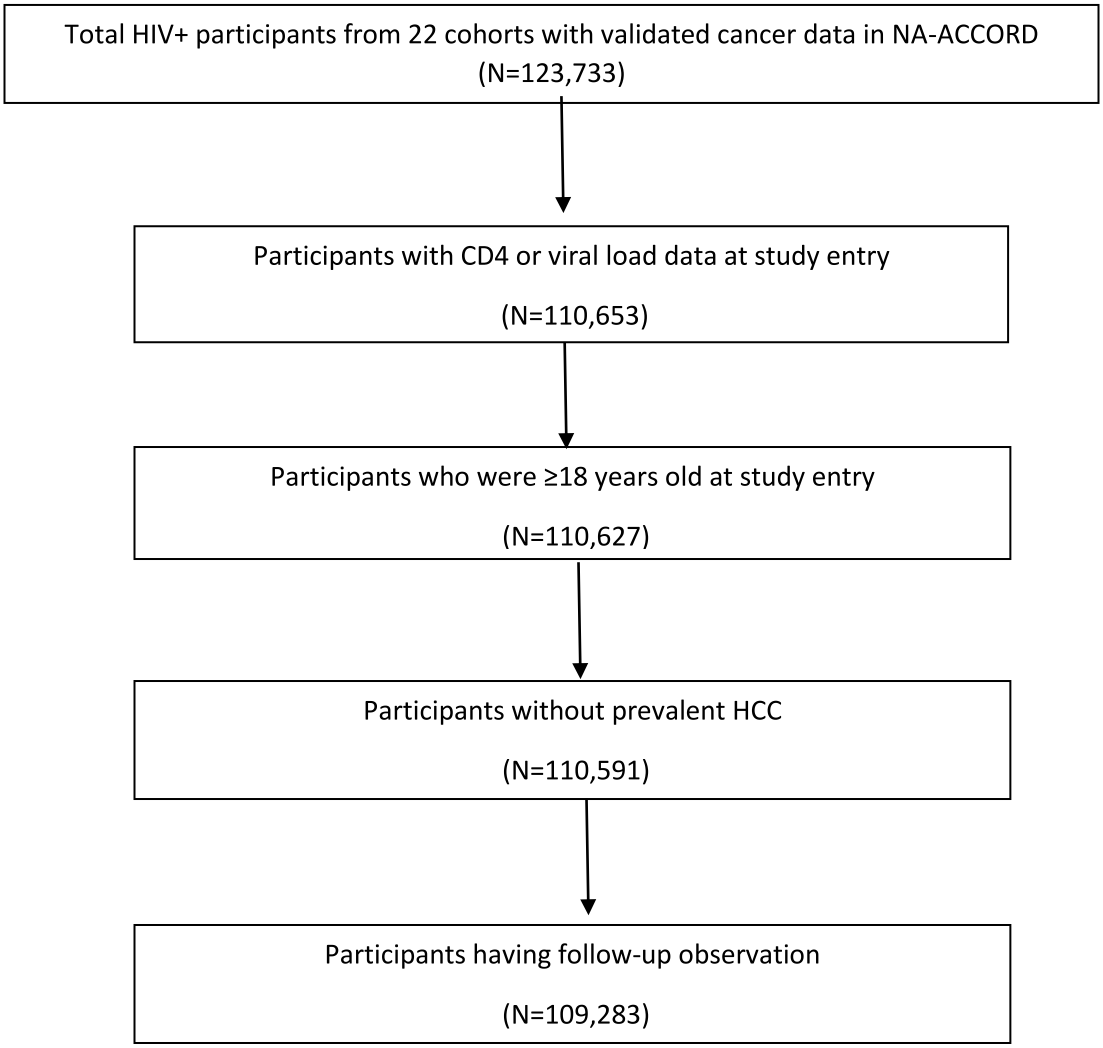

© 2021 Sun J et al. *JAMA Network Open.*

eFigure 2. Crude Incidence Rates of Hepatocellular Carcinoma in NA-ACCORD by cART Eras, 1996-2015.

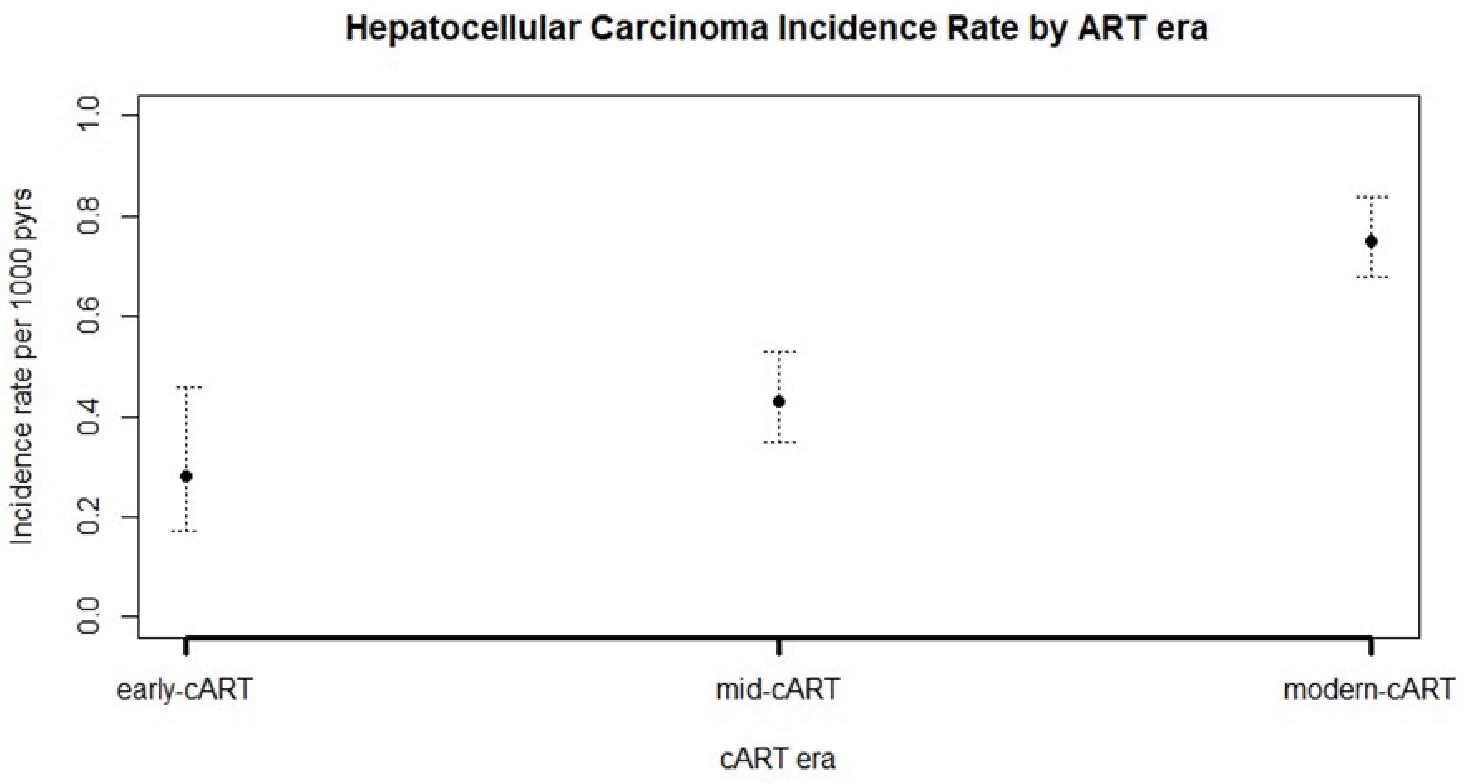

© 2021 Sun J et al. *JAMA Network Open.*

eFigure 3. Crude Cumulative Incidence Function (CIF) of HCC Compared to CIF Incorporating Competing Risks of Deaths Using Fine & Gray Methods, by Calendar Periods.

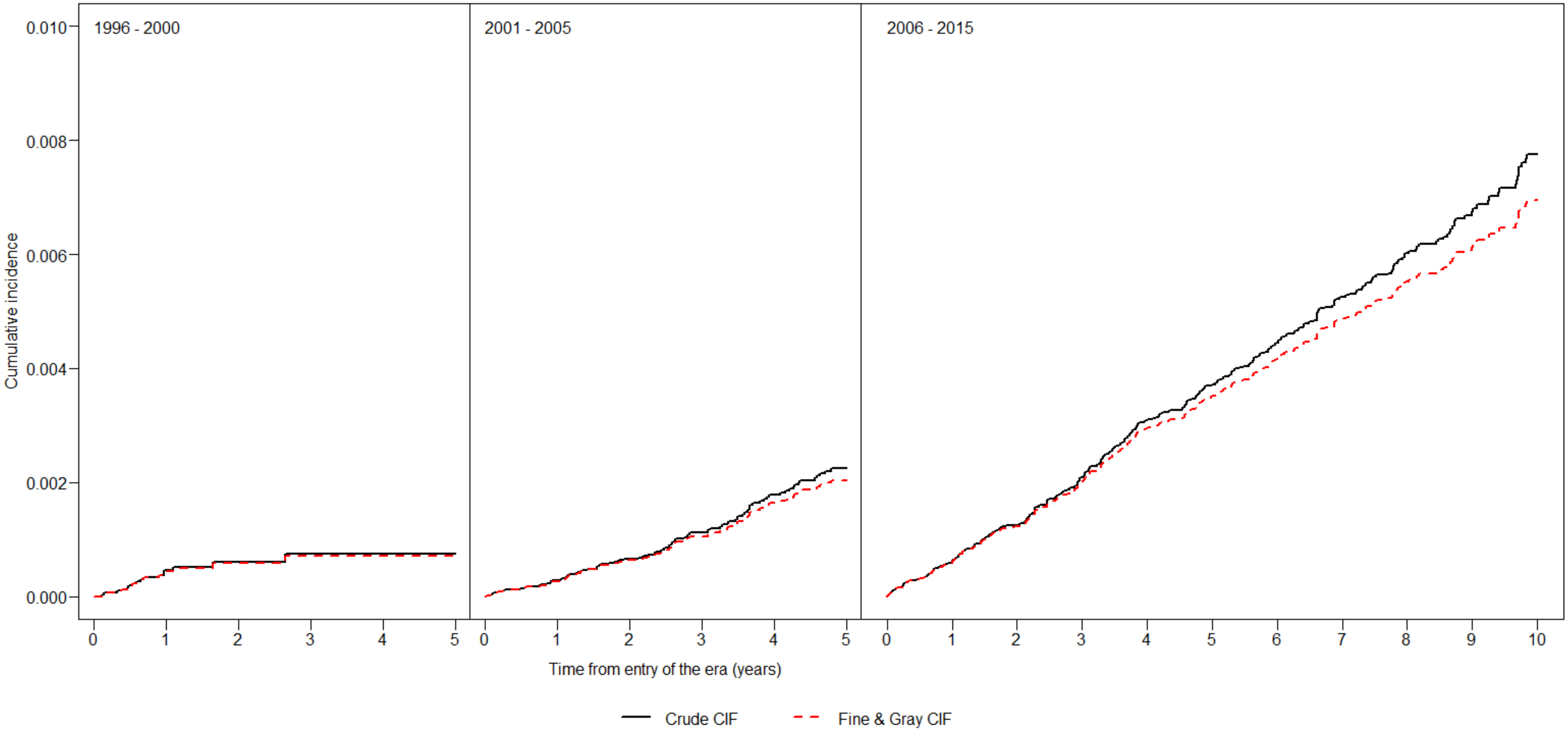

© 2021 Sun J et al. *JAMA Network Open.*

eFigure 4. Distribution of HIV Transmission Risk Group by Viral Hepatitis Coinfection Status in NA-ACCORD.

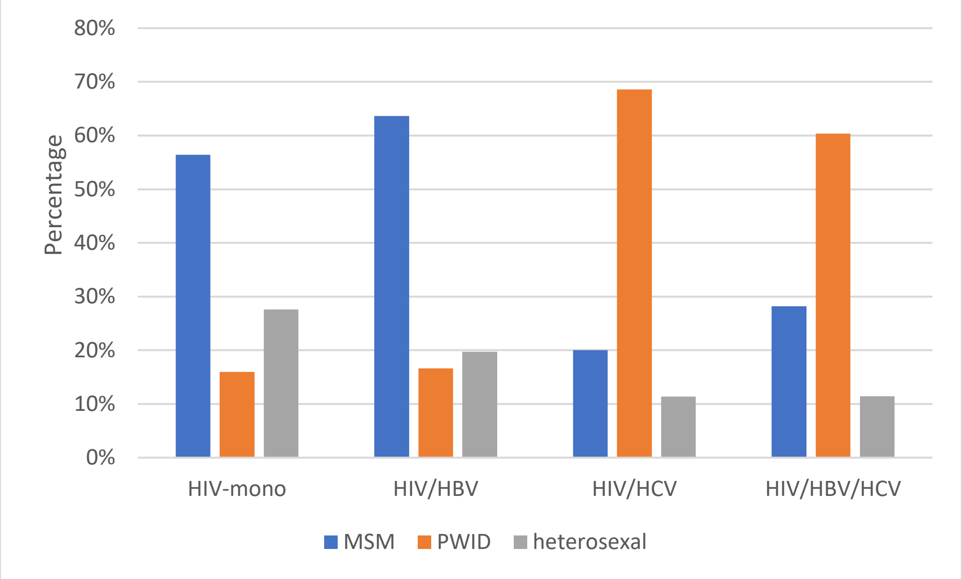

[a]HBV infection was defined by detection of either hepatitis B surface antigen (HBsAg), hepatitis B e antigen (HBeAg), or HBV DNA in serum or plasma at any time during observation. HCV infection was defined by the detection of HCV antibody seropositivity, HCV RNA, or detectable genotype in serum or plasma at any time under observation.

© 2021 Sun J et al. *JAMA Network Open.*

eTable 1. HCC Incidence Rate and Incidence Rate Ratios in NA-ACCORD, by Calendar Period.

				Incidence rate per 1000 pyrs			Unadjusted incidence rate ratio			Adjusted incidence rate ratio		
					95% CI			95% CI			95% CI	
	N	HCC cases	Person years	IR	LL	UL	IRR	LL	UL	aIRR[a]	LL	UL
Early-cART era(1996-2000)	33661	16	57338	0.28	0.17	0.46	ref	n/a	n/a	ref	n/a	n/a
Mid-cART era (2001-2005)	62583	87	203843	0.43	0.35	0.53	1.53	0.90	2.61	1.01	0.59	1.72
Modern-cART era (2006-2015)	87117	348	462260	0.75	0.68	0.84	2.70	1.63	4.45	1.72	1.03	2.87

Note: HCC, hepatocellular carcinoma; IR, incidence rate; IRR, incidence rate ratio; LL, lower limit; UL, upper limit; aIRR, adjusted incidence rate ratio.
[a] Incidence rate ratios adjusted for age (<40, 40-49, 50-59, >=60), sex, race, cohort and viral hepatitis infection

© 2021 Sun J et al. *JAMA Network Open.*

eTable 2. Age Specific HCC Incidence in NA-ACCORD by Calendar Periods.

				Incidence rate per 1000 Pyrs		
					%95 CI	
ART Calendar eras	Age groups	HCC events	Person years	IR	LL	UL
Early-cART era (1996-2000)	<40 years	0	24630	0	.	.
	40 - 49 years	6	21277	0.28	0.13	0.63
	50 - 59 years	8	9103	0.88	0.44	1.76
	>=60 years	2	2328	0.86	0.21	3.44
Mid-cART era (2001-2005)	<40 years	1	53981	0.02	0	0.13
	40 - 49 years	20	80612	0.25	0.16	0.38
	50 - 59 years	48	53809	0.89	0.67	1.18
	>=60 years	18	15442	1.17	0.73	1.85
Modern-cART era (2006-2015)	<40 years	3	82473	0.04	0.01	0.11
	40 - 49 years	32	138248	0.23	0.16	0.33
	50 - 59 years	157	151819	1.03	0.88	1.21
	>=60 years	156	89721	1.74	1.49	2.03
Overall	<40 years	4	161084	0.02	0.01	0.07
	40 - 49 years	58	240136	0.24	0.19	0.31
	50 - 59 years	213	214730	0.99	0.87	1.13
	>=60 years	176	107491	1.64	1.41	1.9

© 2021 Sun J et al. *JAMA Network Open.*

eTable 3. Hepatocellular Carcinoma Incidence Rates and Incidence Rate Ratios in NA-ACCORD Before and After 2006 by Viral Hepatitis Coinfection Groups.

				Incidence rate per 1000 Pyrs			unadjusted incident ratio			Adjusted incident ratio		
					%95 CI			95% CI			95% IC	
**Hepatitis and HIV risk groups**	**ART era**	**HCC events**	**Person years**	**IR**	**LL**	**UL**	**IRR**	**LL**	**UL**	**aIRR[a]**	**LL**	**UL**
Overall	Early + mid-cART era (1996-2005)	103	261181	0.39	0.33	0.48	Ref	.	.	Ref	.	.
	Modern-cART era (2006-2015)	348	462260	0.75	0.68	0.84	1.9	1.53	2.38	1.5	1.16	1.82
HIV monoinfected	Early + mid-cART era (1996-2005)	11	165451	0.07	0.04	0.12	Ref	.	.	Ref	.	.
	Modern-cART era (2006-2015)	26	322437	0.08	0.05	0.12	1.2	0.6	2.45	.82	0.4	1.68
HIV/HCV	Early + mid-cART era (1996-2005)	60	64947	0.92	0.72	1.19	Ref	.	.	Ref	.	.
	Modern-cART era (2006-2015)	226	94361	2.4	2.1	2.73	2.6	1.95	3.45	2	1.51	2.75
HIV/HBV	Early + mid-cART era (1996-2005)	25	17560	1.42	0.96	2.11	Ref	.	.	Ref	.	.
	Modern-cART era (2006-2015)	54	25864	2.09	1.6	2.73	1.5	0.91	2.36	1.3	0.78	2.07
HIV/HBV/HCV	Early + mid-cART era (1996-2005)	3	7082	0.42	0.14	1.31	Ref	.	.	Ref	.	.
	Modern-cART era (2006-2015)	38	8457	4.49	3.27	6.18	11	3.27	34.36	10	3	33.15

[a] incidence rate ratios adjusted for age (<40,40-49,50-59,>=60),sex, race and cohort.

© 2021 Sun J et al. *JAMA Network Open.*

eTable 4. Sensitivity Analysis of Hepatocellular Carcinoma Incidence Rates by Hepatitis C and Hepatitis B and ART Era Controlled for Alcohol and Smoking Behavior.

					Incidence rate per 1000 Pyrs			Unadjusted incidence ratio			Adjusted incidence ratio		
						95% CI			95% CI			95% CI	
	Viral hepatitis co-infection groups*	N	HCC events	Person years	IR	LL	UL	IRR	LL	UL	aIRR[a]	LL	UL
Early-cART era (1996-2000)	Overall	8037	2	19828	0.1	0.03	0.4	n/a	n/a	n/a	n/a	n/a	n/a
	HBV- & HCV-[b]	5293	0	13007	0	0	0.28	ref	n/a	n/a	ref	n/a	n/a
	HCV+ only[c]	2155	0	5443	0	0	0.68	n/a	n/a	n/a	n/a	n/a	n/a
	HBV+ only[d]	375	1	880	1.14	0.16	8.07	n/a	n/a	n/a	n/a	n/a	n/a
	HBV+ & HCV+[e]	214	1	497	2.01	0.28	14.27	n/a	n/a	n/a	n/a	n/a	n/a
Mid-cART era (2001-2005)	Overall	16007	19	51667	0.37	0.23	0.58	n/a	n/a	n/a	n/a	n/a	n/a
	HBV- & HCV-	10362	0	32799	0	0	0.11	ref	n/a	n/a	ref	n/a	n/a
	HCV+ only	3434	12	11232	1.07	0.61	1.88	n/a	n/a	n/a	n/a	n/a	n/a
	HBV+ only	731	5	2294	2.18	0.91	5.24	n/a	n/a	n/a	n/a	n/a	n/a
	HBV+ & HCV+	327	0	1140	0	0	3.24	n/a	n/a	n/a	n/a	n/a	n/a
Modern-cART era (2006-2015)	Overall	26727	45	130121	0.35	0.26	0.46	n/a	n/a	n/a	n/a	n/a	n/a
	HBV- & HCV-	19244	6	90322	0.07	0.03	0.15	ref	n/a	n/a	ref	n/a	n/a
	HCV+ only	4628	18	23423	0.77	0.48	1.22	11.6	4.6	29.1	10.5	4.1	27.2
	HBV+ only	1127	13	5694	2.28	1.33	3.93	34.4	13.1	90.4	32.5	12.2	86.2
	HBV+ & HCV+	397	6	2176	2.76	1.24	6.14	41.5	13.4	129	39.8	12.5	127

Note: HCC, hepatocellular carcinoma; IR, incidence rate; IRR, incidence rate ratio; LL, lower limit; UL, upper limit; aIRR, adjusted incidence rate ratio.

[a] incidence rate ratios adjusted for age (<40, 40-49, 50-59, >=60), sex, race, smoking, alcohol use and cohort;

[b] HBV- & HCV- was defined as HBV negative and HCV negative;

[c] HCV+ only was defined as HCV positive and (HBV negative or HBV not assessed);

[d] HBV+ only was defined as HBV positive and (HCV negative or HCV not assessed);

[e] HBV+ & HCV+ was defined as HBV positive and HCV positive

*HBV infection was defined by detection of either hepatitis B surface antigen (HBsAg), hepatitis B e antigen (HBeAg), or HBV DNA in serum or plasma at any time during observation. HCV infection was defined by the detection of HCV antibody seropositivity, HCV RNA, or detectable genotype in serum or plasma at any time under observation.

© 2021 Sun J et al. *JAMA Network Open.*

eTable 5. Distribution of Age at Hepatocellular Carcinoma Diagnosis and Age of Underlying Population by Viral Hepatitis Coinfection.

Viral hepatitis coinfection groups	Overall		HCC cases	
	N	Median (IQR)	N	Median age (IQR)
**HIV monoinfected**	76897	42 (34-50)	37	61 (53-68)
**HIV/HCV+**	21343	47 (41-52)	286	59 (55-64)
**HIV/HBV+**	6348	42 (36-50)	79	54 (47-58)
**HIV/HBV+/HCV+**	2082	45 (39-50)	41	56 (50-61)
**HBV and HCV not assessed**	2613	42 (35-49)	8	54 (47-56)
**Total**	109283	43 (36-51)	451	58 (54-63)

© 2021 Sun J et al. *JAMA Network Open.*

eTable 6. Incidence Rates and Incidence Rate Ratios of Hepatocellular Carcinoma by CD4 Counts by cART Periods.

					Incidence rate per 1000 Pyrs			Unadjusted incidence ratio			Adjusted incidence ratio		
						95% CI			95% CI			95% CI	
	CD4 groups[b]	N	HCC events	Person years	IR	LL	UL	IRR	LL	UL	aIRR[a]	LL	UL
Early-cART era(1996-2000)	Overall	33661	16	57338	0.28	0.17	0.46	n/a	n/a	n/a	n/a	n/a	n/a
	≤500	23411	14	40799	0.34	0.20	0.58	n/a	n/a	n/a	n/a	n/a	n/a
	>500	8436	0	14182	0.00	0.00	0.26	ref	n/a	n/a	ref	n/a	n/a
	Missing	1814	2	2356	0.85	0.21	3.39	n/a	n/a	n/a	n/a	n/a	n/a
Mid-cART era (2001-2005)	Overall	62583	87	203843	0.43	0.35	0.53	n/a	n/a	n/a	n/a	n/a	n/a
	≤500	39557	52	124627	0.42	0.32	0.55	1.0	0.6	1.5	0.9	0.6	1.4
	>500	17777	27	62433	0.43	0.30	0.63	ref	n/a	n/a	ref	n/a	n/a
	Missing	5249	8	16784	0.48	0.24	0.95	1.1	0.5	2.4	1.0	0.5	2.2
Modern-cART era (2006-2015)	Overall	87117	348	462260	0.75	0.68	0.84	n/a	n/a	n/a	n/a	n/a	n/a
	≤500	48997	223	258035	0.86	0.76	0.99	1.4	1.1	1.8	1.3	1.0	1.6
	>500	29407	96	159457	0.60	0.49	0.74	ref	n/a	n/a	ref	n/a	n/a
	Missing	8713	29	44769	0.65	0.45	0.93	1.1	0.7	1.6	0.7	0.5	1.1

Note: HCC, hepatocellular carcinoma; IR, incidence rate; IRR, incidence rate ratio; LL, lower limit; UL, upper limit; aIRR, adjusted incidence rate ratio.
[a] incidence rate ratios adjusted for age (<40, 40-49, 50-59, >=60 years), sex, race, cohort and viral hepatitis infection
[b] CD4 was assessed at the entry of each era as CD4 closest to the entry within window of 6 months prior to 3 months after the entry date

© 2021 Sun J et al. *JAMA Network Open.*

eTable 7. Incidence Rates and Incidence Rate Ratios of Hepatocellular Carcinoma by CD4 Percentage by cART periods.

					Incidence rate per 1000 Pyrs			Unadjusted incidence ratio			Adjusted incidence ratio		
						95% CI			95%			%95 CI	
	CD4 % groups[b]	N	HCC events	Person years	IR	LL	UL	IRR	LL	UL	aIRR[a]	LL	UL
Early-cART era (1996-2000)	Overall	33661	16	57338	0.28	0.17	0.46	n/a	n/a	n/a	n/a	n/a	n/a
	≤29%	23263	10	39427	0.25	0.14	0.47	0.8	0.2	2.4	0.5	0.2	1.7
	>29%	6973	4	11957	0.33	0.13	0.89	ref	n/a	n/a	ref	n/a	n/a
	Missing	3425	2	5954	0.34	0.08	1.34	1	0.2	5.5	0.6	0.1	3.5
Mid-cART era (2001-2005)	Overall	62583	87	203843	0.43	0.35	0.53	n/a	n/a	n/a	n/a	n/a	n/a
	≤29%	38437	53	124501	0.43	0.33	0.56	0.7	0.5	1.2	0.6	0.4	1
	>29%	13284	27	45979	0.59	0.4	0.86	ref	n/a	n/a	ref	n/a	n/a
	Missing	10862	7	33364	0.21	0.1	0.44	0.4	0.2	0.8	0.4	0.2	0.8
Modern-cART era (2006-2015)	Overall	87117	348	462260	0.75	0.68	0.84	n/a	n/a	n/a	n/a	n/a	n/a
	≤29%	52315	225	282004	0.8	0.7	0.91	1.1	0.9	1.4	0.9	0.7	1.2
	>29%	24200	93	128227	0.73	0.59	0.89	ref	n/a	n/a	ref	n/a	n/a
	Missing	10602	30	52029	0.58	0.4	0.82	0.8	0.5	1.2	0.6	0.4	0.9

Note: HCC, hepatocellular carcinoma; IR, incidence rate; IRR, incidence rate ratio; LL, lower limit; UL, upper limit, aIRR, adjusted incidence rate ratio.
[a] incidence rate ratios adjusted for age (40, 40-49, 50-59, ≥60), sex, race, and viral hepatitis infection.
[b] CD4% was assessed at the entry of each era as CD4% closest to the entry within window of 6 months prior to 3 months after the entry date.

© 2021 Sun J et al. *JAMA Network Open.*

eTable 8. Incidence Rates and Incidence Rate Ratios of Hepatocellular Carcinoma by HIV Viral Load by cART Periods.

					Incidence rate per 1000 Pyrs			Unadjusted incidence ratio			Adjusted incidence ratio		
						95% CI			95%			%95 CI	
	HIV RNA viral load groups[b]	N	HCC events	Person years	IR	LL	UL	IRR	LL	UL	aIRR[a]	LL	UL
Early-cART era (1996-2000)	Overall	33661	16	57338	0.28	0.17	0.46	n/a	n/a	n/a	n/a	n/a	n/a
	<500	4317	1	14187	0.07	0.01	0.5	ref	n/a	n/a	ref	n/a	n/a
	≥500	7268	1	20546	0.05	0.01	0.35	0.7	0	11	1	0.1	16.1
	Missing	22076	14	22605	0.62	0.37	1.05	8.8	1.2	66.8	6.1	0.8	49.3
Mid-cART era (2001-2005)	Overall	62583	87	203843	0.43	0.35	0.53	n/a	n/a	n/a	n/a	n/a	n/a
	<500	24633	39	96738	0.4	0.29	0.55	ref	n/a	n/a	ref	n/a	n/a
	≥500	23707	30	78864	0.38	0.27	0.54	0.9	0.6	1.5	1.1	0.7	1.8
	Missing	14243	18	28241	0.64	0.4	1.01	1.6	0.9	2.8	1.9	1.1	3.3
Modern-cART era (2006-2015)	Overall	87117	348	462260	0.75	0.68	0.84	n/a	n/a	n/a	n/a	n/a	n/a
	<500	56668	219	353162	0.62	0.54	0.71	ref	n/a	n/a	ref	n/a	n/a
	≥500	17569	75	68491	1.1	0.87	1.37	1.8	1.4	2.3	1.8	1.4	2.4
	Missing	12880	54	40607	1.33	1.02	1.74	2.1	1.6	2.9	2.2	1.6	3

Note: HCC, hepatocellular carcinoma; IR, incidence rate; IRR, incidence rate ratio; LL, lower limit; UL, upper limit; aIRR, adjusted incidence rate ratio.
[a] incidence rate ratios adjusted for age (<40, 40-49, 50-59, >=60), sex, race and viral hepatitis infection

[b] HIV RNA viral load was assessed as HIV RNA viral load closest to the date of 2 years prior to HCC diagnosis date or date of 2 years prior to end of follow-update for non-HCC cases within each calendar period

© 2021 Sun J et al. *JAMA Network Open.*

eTable 9. Incidence Rates and Incidence Rate Ratios of Hepatocellular Carcinoma by HIV Transmission Risk Group and Calendar Years

	HIV transmission risk	N	HCC events	Person years	Incidence rate per 1000 Pyrs	Unadjusted incidence rate ratio	Adjusted[a] incidence rate ratio	Adjusted[b] incidence rate ratio
Early-cART era (1996-2000)	MSM	8330	1	19371	0.05 (0.01-0.37)	Ref.	Ref.	Ref.
	PWID	9464	6	13695	0.44 (0.2-0.98)	8.5 (1.0-70.5)	7.7 (0.9-64.2)	9.1 (1.0-84.1)
	Heterosexual	4134	0	9911	0.00 (0.00-0.37)	n/a	n/a	n/a
	Other/unknown	11733	9	14361	0.63 (0.33-1.20)	12.1 (1.5-95.8)	9.4 (1.2-75.4)	10.7 (1.3-86.5)
Mid-cART era (2001-2005)	MSM	17956	14	54911	0.25 (0.15-0.43)	Ref.	Ref.	Ref.
	PWID	14861	37	51872	0.71 (0.52-0.98)	2.8 (1.5-5.2)	2.3 (1.2-4.5)	1.6 (0.7-3.3)
	Heterosexual	9289	1	28967	0.03 (0.00-0.25)	0.1 (0.0-1.0)	0.3 (0.0-2.1)	0.3 (0.0-2.6)
	Other/unknown	20477	35	68094	0.51 (0.37-0.72)	2.0 (1.1-3.7)	1.6 (0.8-3.0)	1.8 (0.9-3.7)
Modern-cART era (2006-2015)	MSM	28656	40	130961	0.31 (0.22-0.42)	Ref.	Ref.	Ref.
	PWID	15143	168	92523	1.82 (1.56-2.11)	5.9 (4.2-8.4)	4.1 (2.8-6.0)	2.0 (1.3-2.9)
	Heterosexual	12996	12	59855	0.2 (0.11-0.35)	0.7 (0.3-1.3)	1.0 (0.5-1.9)	1.2 (0.6-2.3)
	Other/unknown	30322	128	178921	0.72 (0.6-0.85)	2.3 (1.6-3.3)	1.8 (1.2-2.5)	1.8 (1.2-2.7)

Note: HCC, hepatocellular carcinoma; IR, incidence rate; IRR, incidence rate ratio; LL, lower limit; UL, upper limit; aIRR, adjusted incidence rate ratio.
[a] incidence rate ratios adjusted for age (<40, 40-49, 50-59, >=60 years), sex, race and cohort;
[b] incidence rate ratios adjusted for age (<40, 40-49, 50-59, >=60), sex, race, cohort and viral hepatitis co-infection.

© 2021 Sun J et al. *JAMA Network Open.*

eTable 10. Hepatocellular Carcinoma Incidence Rates and Incidence Rate Ratios Before and After 2006 by HIV Transmission Risk Groups

				Incidence rate per 1000 Pyrs			unadjusted incident ratio			Adjusted incident ratio		
					%95 CI			95% CI			95% IC	
**HIV transmission risk groups**	**ART era**	**HCC events**	**Person years**	**IR**	**LL**	**UL**	**IRR**	**LL**	**UL**	**aIRR[a]**	**LL**	**UL**
MSM	Early + Mid-cART era (1996-2005)	15	74282	0.2	0.12	0.33	Ref	.	.	Ref	.	.
	Modern-cART era (2006-2015)	40	130961	0.31	0.22	0.42	1.5	0.84	2.74	1.6	0.85	2.89
IDU	Early + Mid-cART era (1996-2005)	43	65567	0.66	0.49	0.88	Ref	.	.	Ref	.	.
	Modern-cART era (2006-2015)	168	92523	1.82	1.56	2.11	2.8	1.98	3.87	2.7	1.88	3.86
Heterosexual	Early + Mid-cART era (1996-2005)	1	38878	0.03	0	0.18	Ref	.	.	Ref	.	.
	Modern-cART era (2006-2015)	12	59855	0.2	0.11	0.35	7.8	1.01	59.94	7.3	0.92	57.56

[a] incidence rate ratios adjusted for age (<40,40-49,50-59,>=60), sex, race, cohort, and viral hepatitis coinfection groups.

© 2021 Sun J et al. *JAMA Network Open.*
